# Commentary: Development of a Comparative European Orthohantavirus Microneutralization Assay With Multi-Species Validation and Evaluation in a Human Diagnostic Cohort

**DOI:** 10.3389/fcimb.2021.702709

**Published:** 2021-08-04

**Authors:** Jan Clement, Jan Groen, Guido van der Groen, Marc Van Ranst, Piet Maes, Albertus D. M. E. Osterhaus

**Affiliations:** ^1^KULeuven, Rega Institute for Medical Research, Laboratory of Clinical and Epidemiological Virology, Leuven, Belgium; ^2^National Reference Center for Hantavirus, University Hospitals Leuven, Leuven, Belgium; ^3^Laboratory of Immunobiology, Institute of Public Health and Environmental Protection, Bilthoven, Netherlands; ^4^Virology Unit, Institute of Tropical Medicine, Antwerp, Belgium

**Keywords:** rats, Seoul orthohantavirus, Puumala orthohantavirus, haemorrhagic fever with renal syndrome, acute kidney injury, serodiagnosis, biomolecular diagnosis, Netherlands

## Introduction

**Figure 1:**
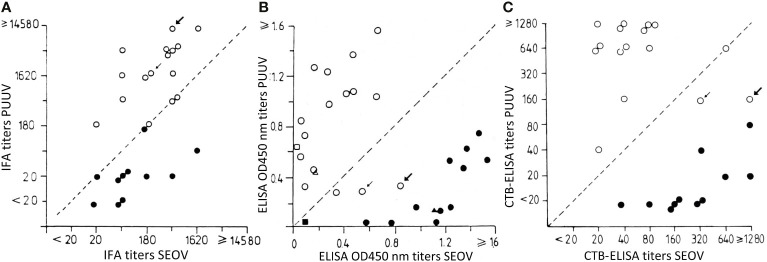
IFA: immuno-fluorescence assay or immuno-fluorescence test (IFT). ELISA, enzyme-linked immunosorbent assay. CTB ELISA, complex-trapping blocking, an inhibition ELISA variant. Closed circles: 1980s laboratory rat-acquired human hantavirus infections. Open circles: non-laboratory rat-acquired or “wild” human hantavirus infections, mostly being 1980s PUUV-induced, except for two “wild” cases (arrows), being 1980s wild rat-induced SEOV infections. Thick arrow: Dutch farmer, thin arrow: Belgian homeless vagabond, both with wild rat-exposure. In these two cases, SEOV infection was missed in IFA, showing surprisingly high cross-reacting PUUV titers **(A)**, but confirmed, albeit with lower titers, in ELISA **(B)**, and unmistakably ascertained in CTB-ELISA **(C)**. Consequently, these two cases would have been mistaken for PUUV infections, if relying only on IFT/IFA. Adapted from Groen et al., 1991. Copyright ^©^ 1991 Wiley‐Liss, Inc., A Wiley Company.

In this otherwise excellent article (Hoornweg et al., 2020) cited “Three human SEOV infections were confirmed, of which one was previously described as the first [2018] proven SEOV case in The Netherlands, based on IFT [immuno-fluorescence test] serology and an epidemiological link to SEOV RNA-positive [wild] rats) (Swanink et al., 2018), and now confirmed by comparative VNT [virus neutralization test].” This is historically incorrect: 27 years before Swanink et al, Groen et al. already reported wild rat-induced human SEOV infections in The Netherlands, moreover, in the same Dutch Institute of Public Health and Environmental Protection, Bilthoven, and with the same IFT (or IFA) technique (Groen et al., 1991). In addition, this 1991 report compared 14 non-laboratory cases of hemorrhagic fever with renal syndrome (HFRS), caused by the common European arvicolid orthohantavirus Puumala (PUUV), to 13 cases infected by the then rare (at least in early 1990s Western literature) orthohantavirus Seoul (SEOV), a juxtaposition in one single paper of two different local Dutch pathogenic orthohantaviruses, thus constituting at that moment the earliest such confrontation in nascent Western hantavirus literature (**Figure 1**). Moreover, not only 11 Dutch and/or Belgian laboratory rat-induced early 1980s SEOV-HFRS cases were detected, but also two wild rat-induced cases (arrows in **Figure 1**), thereby scoring yet another “first” in western hantavirus literature.

## Historic Reminder

In fact, Dutch hantaviral infections were described already much earlier in four laboratory personnel, in four Lou/M (from “Louvain,” Belgium) laboratory rats, and in four leptospirosis-suspected cases without previous laboratory contacts (Osterhaus et al., 1984). However, for this preliminary pioneer screening, the Korean prototype Hantaan orthohantavirus (HTNV) 76-118 was used, the only hantaviral antigen available at that moment. In IFT, murid HTNV 76-118 strongly cross-reacts with equally murid SEOV, and also, mostly to a lesser degree, with arvicolid PUUV. Of note, these same IFT cross-reactions allowed, from the early 1980s onward, the start of surprisingly efficient serodiagnosis for Western-European HFRS, despite being predominantly PUUV-induced indeed (Clement and van der Groen, 1987), as again confirmed in the 1991 report (**Figure 1**).

In contrast, but often forgotten, the earliest publications of seroproven HFRS in four West-European countries were about SEOV laboratory outbreaks, not about PUUV-induced “wild” cases: a first SEOV laboratory outbreak in Belgium was reported in 1983 (Desmyter et al., 1983), followed by France (Dournon et al., 1984), the UK (Lloyd et al., 1984), and The Netherlands (Osterhaus et al., 1984), simply because of continued import of SEOV-infected Wistar rats from a Brussels immunological laboratory, at a time when SEOV infection was not known, nor suspected, to be present in asymptomatic rats. Remarkably, clinical characteristics of three hospitalized technicians (one of which needed acute hemodialysis) from this Brussels laboratory were described already in 1979 (van Ypersele de Strihou, 1979), meaning 3 years before the earliest English publication of this novel rat-borne pathogen itself, isolated in 1980 by Ho-Wang Lee in Seoul, South-Korea (Lee et al., 1982). The renal presentation in these three cases was intriguing, because it consists of acute kidney injury (AKI), together with massive, even nephrotic-range proteinuria, and microhematuria, all, however, rapidly and surprisingly self-remitting within 2 to 3 weeks. In retrospect, this 1979 description fulfilled all current criteria of COVID-19–induced AKI, another emerging zoonosis with likewise a preceding pro-inflammatory “cytokine storm” (Karras et al., 2021). The same rapid self-remission within 2 to 3 weeks of all clinical parameters was already described before (Clement, 2015; Clement et al., 2019).

In pioneer IFT times, the former prudent circumscription of worldwide omnipresent rat-borne SEOV pathogen was “HTNV-like” (LeDuc et al., 1986; Clement, 2015; Clement et al., 2019). Nevertheless, in the Groen et al. (1991) report, cross-reactions were circumvented by a (then) novel inhibition ELISA variant, called complex-trapping blocking (CTB) ELISA. This CTB assay proved to be faster, more sensitive, and giving even less cross-reactions than most other ELISAs (Groen et al., 1989; Groen et al. 1991). Indeed, Hoornweg et al. admitted that two Dutch SEOV-HFRS cases were missed by classic 2013 IFT/ELISA screening, an inconvenience probably avoidable with CTB-ELISA, as performed three decades before in the same Bilthoven Institute. Moreover, Groen et al. found markedly higher PUUV than SEOV IFT titers in two wild rat-infected cases (**Figure 1A**), a highly unusual feature for cross-reactions, potentially meaning an implicit warning for regions like Finno-Scandia, where sero-diagnoses until now often rely on assays containing only PUUV antigens, considered (wrongly) for decades as the sole local hantaviral pathogen. Of note, not a single SEOV-HFRS case was reported so far from Finno-Scandia, despite demonstration, dating from 1989, of SEOV infection in local rats (LeDuc et al., 1986). This situation results from an almost complete loss of scientific interest in the West for SEOV-HFRS in the 1990s (Clement et al., 1997). Virologists finally returned their attention to this topic, only when a particularly severe English HFRS case, finally linked to SEOV-infected pet rats, was published in 2013, hereby heralding a so-called new problem of SEOV infections in pet and feeder rats and their owners/breeders (Taori et al., 2013).

## Conclusion

When scientists can use the perfect biomolecular tool today (RT-PCR), enabling them pinpointing exactly the causative pathogen, they should not disdain altogether the work of pioneers, often reaching the same conclusions with (then) innovative, but less perfect serotechniques, such as the use of monoclonal antibodies and/or CTB ELISA. Disregarding such results, because “*never confirmed by either RT-PCR or VNT*” (Hoornweg et al., 2020) is not realistic, because such techniques were simply not or barely available over three decades ago. Moreover, the “gold standard” RT-PCR is often negative after admission for suspected SEOV-HFRS (Taori et al., 2013, Swanink et al., 2018, Clement et al., 2019), because of the short-lived viremia in humans. In contrast, SEOV viremia in infected rats lasts probably life-long, making them not only vectors but also SEOV reservoirs everywhere, particularly in urban surroundings worldwide. Consequently, it remains much more convenient to demonstrate biomolecularly hantavirus infections in (asymptomatic) carrier rodents than in (even symptomatic) patients. Finally, RT-PCR was negative, and VNT was not applied either by Swanink et al., for claiming their “first” wild rat-induced human SEOV infection in the Netherlands (Swanink et al., 2018).

## Author Contributions

JC conceived the idea and wrote the text. JG and AO performed the described serologic tests in 1991, in RIVM Institute Bilthoven. GG performed serological confirmation of the first HFRS cases in Belgium, Germany and the Netherlands. MR and PM supervised the texts. All authors contributed to the article and approved the submitted version.

## Conflict of Interest

The authors declare that the research was conducted in the absence of any commercial or financial relationships that could be construed as a potential conflict of interest.

## Publisher’s Note

All claims expressed in this article are solely those of the authors and do not necessarily represent those of their affiliated organizations, or those of the publisher, the editors and the reviewers. Any product that may be evaluated in this article, or claim that may be made by its manufacturer, is not guaranteed or endorsed by the publisher.
